# Risk and protective factors for resilience among adolescents and young adults with beta-thalassemia major

**DOI:** 10.1186/s40359-023-01268-2

**Published:** 2023-08-11

**Authors:** Masoume Rambod, Saeed Hamidizadeh, Mohammad-Rafi Bazrafshan, Ali Mohammad Parviniannasab

**Affiliations:** 1 Community Based Psychiatric Care Research Center, School of Nursing and Midwifery, Shiraz University of Medical Sciences, Shiraz, Iran; 2 Assistant Professor of Nursing Education, Department of Nursing, School of Nursing and Midwifery, Shiraz University of Medical Sciences, Shiraz, Iran; 3 Department of Nursing, School of Nursing, Larestan University of Medical Sciences, Larestan, Iran

**Keywords:** Resilience, Beta-thalassemia major, Social support, Hope, Uncertainty, Coping, Mediating effect

## Abstract

**Background:**

Resilience is the ability to overcome adversity in response to a potentially traumatic event. It can relieve people’s discomfort and build personal capacity when facing a stressful situation such as beta thalassemia major. Resilience is a complex and multidimensional concept and is influenced by protective and risk factors. Therefore, the aims of the present study were to (1) investigate the relationship between protective (social support and hope) and risk (uncertainty and defensive coping) factors with resilience and (2) examine the mediating role of courageous coping between these protective and risk factors in resilience.

**Methods:**

This descriptive-analytical study was performed on 312 adolescents and young adults with beta-thalassemia major aged 12–24 years; they were selected using purposeful sampling from two different outpatient thalassemia clinics in the south of Iran. Data were collected in a face-to-face survey using Zimmet Multidimensional Scale of Perceived Social Support, Herth Hope, Stewart Uncertainty in Illness scale, Jalowiec Coping, and Connor–Davidson resilience Scale from April 2022 to November 2022. The collected data were analyzed using descriptive tests, Pearson correlation, and a structural equation model.

**Results:**

According to the main findings of mediation analysis, courageous coping partially mediated the relationship between social support and resilience [(β = 0.042; 95% BC CI (0.003, 0.131)] and fully mediated the relationship between hope and resilience [(β = 0.166; 95% BC CI (0.031, 0.348)]. In other cases, uncertainty and defensive coping had a direct and indirect effect on resilience, respectively.

**Conclusion:**

Based on these results, health professionals and healthcare policymakers should consider this mediator in developing programs to improve resilience. Also, the use of courageous coping could modulate the effect of defensive coping on resilience. Therefore, teaching the use of courageous coping can play an important role in improving resilience.

## Background

Thalassemia major is the most severe form of beta-Thalassemia and a public health concern worldwide [1]. In this disease, deficiency in the production of beta protein in hemoglobin leads to threatening anemia, and the life of a person becomes dependent on regular blood transfusions and constant medical care [2, 3]. The prevalence of beta-thalassemia major (BTM) is usually high in the Mediterranean region, the Middle East, and Southeast Asia [4]. Iran is a Middle Eastern country located in the thalassemia belt [5]. The prevalence of thalassemia in Iranian population is about 4%. it is estimated that there are about 2–3 million carriers of beta-thalassemia major and 25,000 patients in Iran [6].

Thalassemia affects bio-psycho-social health, which can lead to complications such as severe anemia, hepatosplenomegaly, bone disorders especially in the face, growth failure, heart failure, cardiac arrhythmia, infection, late puberty, and red blood cell microcytosis and hypochromia [7]. Patients with BT require frequent blood transfusions to ensure survival, but these interventions increase iron absorption, leading to iron overload. Excess iron causes severe adverse effects in various organ functions, including heart failure and endocrine abnormalities such as diabetes mellitus, hypogonadism, hypothyroidism, hypoparathyroidism, and growth hormone deficiency [8]. Following a mental health disorder, patients may experience anxiety, depression, anger dysregulation, hopelessness, low self-esteem, isolation, and premature death [9, 10]. Therefore, these patients must develop the ability to adapt and perform optimally to overcome emotional and psychological problems [11]. This ability is explained through the concept of resilience.

Resilience is a positive adaptation in the face of adversity which helps maintain one’s physical and psychological well-being [12, 13], particularly in vulnerable children [14]. Considering the complex and multidimensional concept of resilience, it is necessary to examine other concepts of health and its prerequisites [15, 16]. resilience is a dynamic concept; based on the Resilience in Illness Model (RIM), it is affected by both protective and risk factors [17]. If protective factors cannot withstand adverse events, resilience will decrease; on the contrary, resilience will sustain balance and even improve. The results of previous studies showed social support and hope as important protective factors of resilience [18, 19]. Perceived social support can effectively regulate a person’s mental pressure and improve their physical and mental health, and in turn develop resilience in an individual [20]. Snyder et al.’s (1991) hope theory defines hopes as human strengths that are determined by one’s ability to set clear goals, expand specific strategies to achieve goals (paths), and maintain motivation to use the existing strategies [21]. Previous studies have reported that hope is related to health indicators such as coping and resilience [22, 23].

According to Haase’s model, uncertainty and defensive coping are known as the risk factors that can negatively affect resilience [19]. Lazarus and Folkman (1966) describe coping as cognitive and behavioral efforts that a person applies to manage stress [24]. A previous study report that defensive coping adversely affects resilience; of course, people can use courageous coping to buffer the effect of defensive coping on resilience [14, 25]. Uncertainty acts as a main source of stress for patients affected by chronic disease [26]. Uncertainty not only deteriorates psychological problems such as psychological distress, anxiety, and impaired sense of mastery, but also creates negative coping strategies and decreases resilience [26, 27].

Resilience in illness model on chronic patients showed that courageous coping mediated the relationship between protective and risk factors and resilience [17]. Although research has demonstrated the effects of these concepts on resilience in other chronic diseases, little knowledge is available on adolescents and young adults with BTM. Given that the concept of resilience is different according to the social and cultural context, people’s characteristics, and different situations [13, 28], considering the chronicity of diseases such as thalassemia, successful adaptation to this disease is of great importance. Therefore, using a model to design nursing interventions can be the basis for creating resilience to overcome these challenges. On the other hand, the results of this research can help health service providers to increase the capacity of resilience and its development in adolescents by understanding the nature and structure of resilience.

**Fig. 1:**
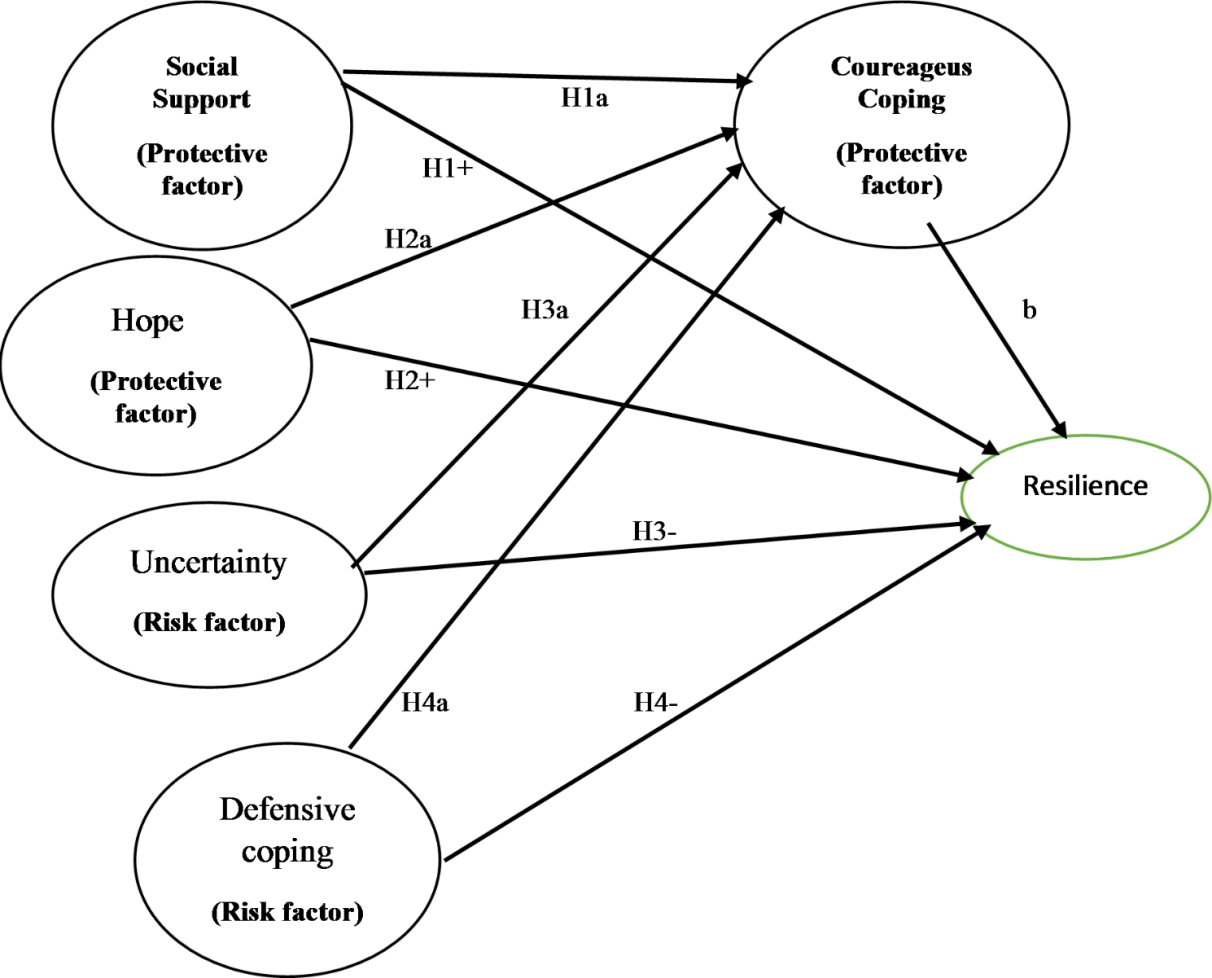
Theoretical framework

Based on previous studies, we hypothesized that (1) protective factors (social support and hope) are positively related to resilience, (2) risk factors (uncertainty and defensive coping) are negatively correlated with resilience, and (3) courageous coping mediates the relationship between protective and risk factors with resilience (**See** Fig. 1).

## Methods

### Study design, setting and participants

This descriptive-analytical study was performed on 312 adolescents and young adults with BTM aged 12–24 years, who were selected using purposeful sampling from two thalassemia clinics (Larestan and Shiraz) affiliated to the universities of medical sciences in Fars province in the south of Iran. Data were collected in a face-to-face survey from April 2022 to November 2022. Inclusion criteria were age between 12 and 24 years old, definitive diagnosis of beta-thalassemia major, regular blood transfusions, the ability to read and write in Persian, and willingness to participate in the study. The exclusion criteria included confirmed mental illness and incomplete questionnaire. According to the study carried out by Wolf et al. (2012), in the use of structural equation modeling, the sample size is calculated according to the estimation of the free parameters in the model, so that for each free parameter, 5 to 10 samples (observations) are needed. Since we had 53 free parameters in the model, a sample size between 265 and 530 was estimated to be sufficient [29]. Due to the attrition rate of 10%, the sample size expanded to 326. Finally, 14 questionnaires were incomplete and were excluded. Therefore, a total of 312 questionnaires were valid for analysis.

### Measures

The Connor–Davidson Resilience Scale (CD-RISC): Resilience is defined as ‘‘the process of adapting well in the face of adversity, trauma, tragedy, threats or even significant sources of stress [13]. This scale includes 25 items and is divided into five domains: competency, tolerance of negative effect, positive acceptance of change, control, and spiritual influences [13]. Each item is scored using a 5-point Likert scale ranging from 0 = never to 4 = almost always. The reliability of CD-RISC (original version) was tested in the general population (Cronbach’s α = 0.89) [30]. The Cronbach’s α of the CD-RISC in the current study was 0.81.

The Multidimensional Scale of Perceived Social Support (MSPSS): Social support is described as “an exchange of resources between at least two individuals perceived by the provider or the recipient to be intended to enhance the well-being of the recipient” [31]. This scale was developed by Zimmet et al. (1988) and contains 12 items, which are divided into three domains, namely family, friends, and special person (significant other). The three subscales of family, friend, and significant others’ perceived social support consist of four items, and each item is scored using a 5-point Likert scale ranging from 1 = very strongly disagree to 5 = very strongly agree. Cronbach’s α values for its three components and the whole scale were 0.88, 0.87, 0.91, and 0.85, respectively [31]. The Cronbach’s α estimated in the current study was 0.81, 0.79, 0.76, and 0.80 for its three components and the whole, respectively, in the Persian version.

The Jalowiec Coping Scale (JCS): The JCS is based on the study carried out by Lazarus and Folkman [24]. This scale is classified into eight subscales, including confrontive, evasive, optimistic, fatalistic, emotive, palliative, supportive, and self-reliant. In the present study, due to the use of Haase’s model as the framework of the present study, only the subscales of confrontive, optimism, and supportive with 24 items (as courageous coping) and emotional and avoidance coping with 18 items (as defensive coping) were used. Accordingly, courageous coping is a constructive strategy with a positive outlook on illness-related situations that is achieved by using self-management behaviors, confrontation with the situation, and seeking supportive resources from health care providers, family, and friends. Conversely, defensive coping is a strategy that a person uses in times of tension or threat through emotional and avoidant coping in response to distress caused by the symptoms and complications of the disease. All items are rated on a 4-point Likert scale (ranging from 0 = never used to 3 = often used). The total score ranges from 0 to 180, with higher scores indicating greater use of the mentioned coping style. The values of Cronbach’s α from a study on heart transplant patients were 0.81, 0.78, 0.63, and 0.93 for confrontive, optimistic, supportive approaches, and JSC total use score, respectively [32]. Cronbach’s α coefficient was 0.89 in the Persian version [33]. The Cronbach’s α in the current study was 0.82, 0.83, and 0.85 for its two components and the total, respectively.

The Herth Hope Index (HHI): Hope is defined as a coping strategy or the psychosocial internal resources necessary to maintain a positive outlook in the face of adversity and stress [34]. The HHI contains 12 items with three dimensions of temporality and future (4 items), positive readiness and expectancy (4 items), and interconnectedness with self and others (4 items). Each item is scored based on a 4-point Likert-type scale ranging from 0 = strongly disagree to 3 = strongly agree. The total score ranges from 0 to 36, with higher scores indicating a higher level of hope. The Cronbach’s α value for the 12-item HHI was 0.97, representing an acceptable internal consistency [34] Cronbach’s α coefficient was 0.84 in the Persian version [35], and it was 0.78 in this study.

The Uncertainty Scale: Uncertainty is identified when the patient cannot correctly understand the consequences of disease-related events or predict the outcome of the disease [36].This scale consists of 22 items and 3 subscales: “not knowing” (4 item) “not being able to predict” (12 item), and “not being sure what things meant “(6 item). Items are scored based on a 5-point Likert scale ranging from 1 = strongly disagree to 4 = strongly agree. The coefficient of Cronbach’s α in the original version was 0.94 [26]. The coefficient of Cronbach’s α was 0.89 for the overall scale in the Persian version [37]. The Cronbach’s α in the current study was 0.85.

### Ethical consideration

The present study was approved by the local Ethics Committee of Larestan University of Medical Sciences, Larestan, Iran (ethical code: IR.LARUMS. REC.1402.010). Written informed consent was obtained from all the participants. In the case of underage adolescents (< 18 years), informed consent was obtained from their parents. All participants were informed of the aim and methods of the study; they were also assured about their anonymity and confidentiality of data.

### Data analysis

Socio-demographic variables were described using numbers, percentages, and mean ± Sd. Pearson correlation analysis was used to examine the relationships among the study variables. These analyses were conducted using SPSS v 0.24.0 software. The structural equation model (SEM) using AMOS 24.0 with maximum likelihood estimations was used to test the hypothesized model. Multiple fit indices were frequently used to evaluate the model, including the chi-square/degrees of freedom ratio (χ2/df), root mean square error of approximation index (RMSEA), the standardized root mean square residual (SRMR), goodness-of-fit index (GFI), adjusted goodness of-fit index (AGFI), normed fit index (NFI), comparative fit index (CFI), and Tucker–Lewis Index (TLI). In the present study, the following criteria were used to determine whether the model was fit: χ2/df < 3.00, RMSEA < 0.05, SRMR *<* 0.05, GFI > 0.90, AGFI > 0.90, NFI > 0.90, CFI > 0.90, and TLI > 0.90 [38]. Finally, to determine the mediating role of courageous coping, we used the bootstrapping method with 5000 repetitions and the bias-corrected bootstrap 95% confidence interval (CI); if the confidence intervals did not contain the value of zero, it showed statistical significance. The significance level was set at 5% (*p* < 0.05).

## Results

### Descriptive statistics and correlations

The mean (SD) of the participants’ age was 17.56 (2.94) years old. The majority of the participants were female (244, 78.2%). Almost all the participants received blood transfusion twice a week (139, 44.6%), and 36 (11.5%) of them had undergone splenectomy (Table 1). The results of the Pearson correlation between the study variables are shown in Table 2. Hope, social support, and courageous coping were positively and significantly correlated with resilience (*r* = 0.153, *r* = 0.158, *r* = 0.193, *p <* 0.01, respectively). Uncertainty was negatively and significantly correlated with resilience (*r* = 0.243, *p* < 0.01); although, there is no significant correlation between defensive coping and resilience (*r = −* 0.033, *p* = 0.563). (Table 2).

**Table 1:** Demographic characteristics of the participants (N = 312)

Characteristic	Mean ± SD	Frequency	Percentage
Age (years)	17.56 ± 2.94		
12–17		148	47.4%
≥18		164	52.6%
Gender			
Female		244	78.2%
Male		68	21.8%
Splenectomy			
Yes		36	11.5%
No		276	88.5%
Number of blood transfusions			
Twice a week		139	44.6%
Three times a week		105	33.7%
Once a month		68	21.8%

**Table 2:** Correlations, means and standards deviations of study variables

	Number of items	Mean ± SD	1	2	3	4	5	6
1. Hope	12	27.13 ± 5.03	1					
2. social support	12	41.98 ± 8.13	0.064	1				
3. Courageous coping	24	47.60 ± 10.23	0.316**	0.316**	1			
4. Defensive coping	18	26.52 ± 9.23	− 0.031	0.034	0.227**	1		
5. Uncertainty	21	34.51 ± 8.71	0.001	0.041	0.018	-0.20	1	
6. Resilience	25	80.69 ± 10.58	0.153**	0.158**	0.193**	− 0.033	-0.243**	1

The structural model of resilience: The structural equation model (SEM) was performed to measure the relationship between hope, social support, uncertainty, and defensive coping with resilience. First, the data were checked for missing values, outliers, and normal distribution. Any person who did not answer > 15% of the questionnaire items was excluded from the analysis; for the questionnaire data in which the missing value analysis was < 15%, missing data was completely at random (MCAR test Little: Chi-Square = 430.463, df = 413, *p* = 0.267). A non-significant result in Little’s test indicated that there was no pattern in the missing data. Mean imputation was used to replace the missing questionnaire data. Outlier detection showed that five univariate and four multivariate outliers were removed. Skewness and kurtosis were used to determine normality. We tested the normality using skewness (-3, + 3) and kurtosis (-7, + 7), which was established [39]. In the next step, confirmatory factor analysis was performed to assess the measurement model by examining the relationship between observed variables and latent constructs. In general, factor loadings of 0.40 and above are considered satisfactory [40]. The results showed that in the hope measurement model, 3 items had a factor load of less than 0.4, which were removed from the final analysis. Also, in measuring the courageous coping measurement model, 4 items had a factor load below 0.4; they were removed as well. In the examination of other dimensions, none of them had a factor load below 0.4. According to, after eliminating items with low factor loadings -less than 0.40- The fit indices were calculated for the structural model. In the final model, multiple fit indices were acceptable: χ2/*df* = 1.32, RMSEA = 0.028, SRMR = 0.039, GFI = 0.946, AGFI = 0.926, NFI = 0.916, CFI = 0.982, and TLI = 0.978. (Table 3). The social support positively affected resilience (β = 0.21, *t* = 2.58, *p* = 0.010), confirming Hypothesis (1) Hope positively affected resilience (β = 0.28, *t* = 2.61, *p* = 0.009), confirming Hypothesis (2) Uncertainty negatively affected resilience (β =-0.45, *t*=-4.49, *p* = < 0.001), confirming Hypothesis (3) Finally, the relationship between defensive coping and resilience was not significant (β = 0.025, *t*=-0.47, *p* = 0.638), rejecting Hypothesis (4) (Table 4).

**Table 3:** Model fit indicators

Indicator	Value	acceptable Limitation	Ideal Limitation
χ2/df	1.32	<5	<3
RMSEA (Root Mean Square Error Approximation)	0.028	< 0.08	< 0.05
SRMR (the standardized root mean square residual)	0.039	< 0.08	< 0.05
GFI (Goodness of Fit Index)	0.946	>0.90	>0.95
AGFI (Adjusted Goodness of Fit Index)	0.926	>0.80	>0.90
NFI (Normed Fit Index)	0.916	>0.90	>0.95
CFI (Comparative Fit Index)	0.982	>0.90	>0.95
TLI Tucker–Lewis Index	0.978	>0.90	>0.95

**Table 4:** Structural model and path coefficients (n = 312)

Effects	paths	Coefficient	SE	t	p	Result
**Total Effects** (Without accounting the mediator)	H1: SS–> Resilience	0.213	0.083	2.58	0.010^**^	Accepted
	H2: Hope ◊Resilience	0.283	0.105	2.61	0.007**	Accepted
	H3: UC ◊Resilience	-0.458	0.102	-4.49	< 0.001***	Accepted
	H4: DC◊ Resilience	0.002	0.035	0.061	0.951^ns^	rejected

***p < 0.001, **p < 0.01, ns: not significant

### The mediating role of courageous coping

**Fig. 2:**
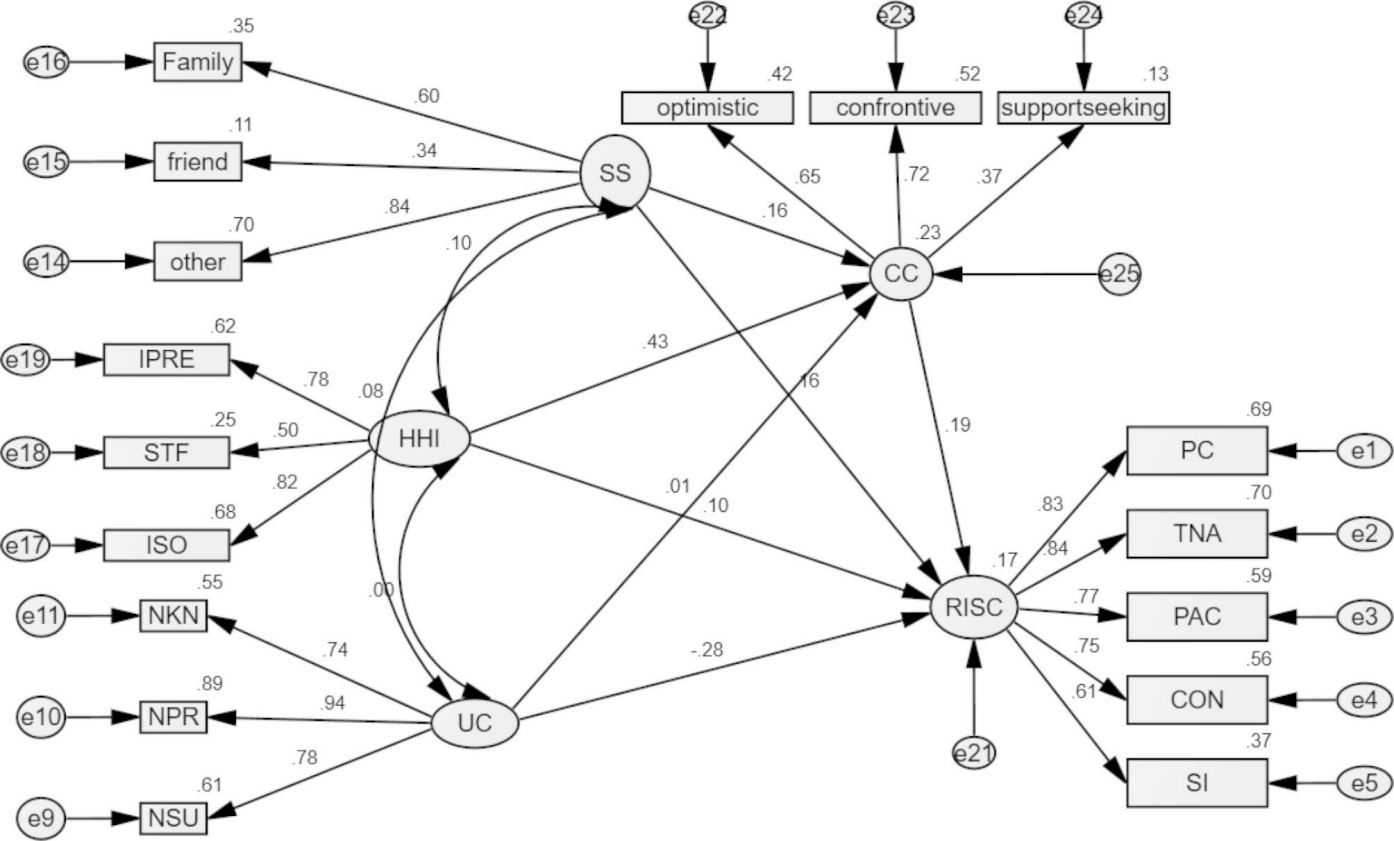
Mediation of courageous coping

For the final hypothesis, we used the bias-corrected bootstrap 95% confidence interval based on 5,000 bootstrapping to test the mediating effect [41]. Social support had an indirect effect on resilience through courageous coping [(β = 0.042; 95% BC CI (0.003, 0.131)]. Additionally, social support had direct effects on resilience through courageous coping [(β = 0.15; 95% BC CI (0.020, 0.374)], indicating the partial mediation effects of courageous coping (H1ab). The hope had an indirect effect on the resilience through courageous coping [(β = 0.166; 95% BC CI (0.031, 0.348)]. However, the direct effect of hope on resilience through courageous coping was not statistically significant (β = 0.130, p = 0.358), indicating the full mediation effects of courageous coping (H2ab). The indirect effect of uncertainty on resilience was not significant via courageous coping [(β = 0.005; 95% BC CI **(**-0.051, 0.071**)]**. Although the direct effect of uncertainty on the resilience was significant [(β=-0.463, *p =* 0.001), uncertainty showed only a direct effect on resilience (H3ab). The indirect effect of defensive coping on the resilience through courageous coping was significant [(β = 0.029; 95% BC CI (0.001, 0.092)]. Although the direct effect of defensive coping on the resilience was not significant (β=-0.041, *p* = 0.105), defensive coping showed only an indirect effect on resilience (H4ab) (Table 5; Fig. 2).

**Table 5:** Results of mediation testing: bootstrap analysis

Mediation Hypothesis		Effect	SE	p	Boot LLCI	Boot ULCI	result
SS◊CC◊Resilience H1ab	**Indirect effect** via courageous coping	0.034	0.028	0.048*	0.001	0.122	Partial Mediation
	**Direct effect** (With accounting the mediator)	0.176	0.084	010^**^	0.039	0.360	
Hope ◊CC◊Resilience H2ab	**Indirect effect** via courageous coping	0.137	0.071	0.016*	0.023	0.300	Full Mediation
	**Direct effect** (With accounting the mediator)	0.162	0.149	0.276^ns^	-0.188	0.471	
UC ◊CC◊Resilience H3ab	**Indirect effect** via courageous coping	0.002	0.025	0.851	− 0.052	0.001	Direct Effect
	**Direct effect** (With accounting the mediator)	-0.459	0.143	0.001^***^	-0.756	-0.184	

***p < 0.001, **p < 0.01, *p < 0.05, ns: not significant

Abbreviations: SS = Social support; UC = Uncertainty; CC = Courageous coping; DC = Defensive coping; SE = Standard error; LLCI = lower limit of confidence interval; ULCI: upper limit of confidence interval

Abbreviations: SS = Social support; Herth Hope Index; UC = Uncertainty; CC = Courageous coping; DC = Defensive coping; Resilience Scale; PC = personal competence = tolerance of negative affect; PAC = positive acceptance of change; CON = control; SI = spiritual influence; IPRE = inner positive readiness and expectancy; STF = sense of temporal and future; ISO = interconnectedness with self and others; NKN = Not Knowing; NPR = Not Being able to predict; NSU = Not Being sure what things meant

## Discussion

Regarding the first hypothesis, the results indicated that social support was positively and significantly related to resilience. In several studies, the results showed a positive and direct association between social support and resilience [42, 43]. In the study of Augustin et al. (2019), the findings indicated the positive effect of social support on resilience [44]. In other studies, the roles of both family [45] and friends were related to high levels of resilience [46]. It seems that receiving social support as a protective factor plays an important role against and having strong sources of social support somehow plays an important role in increasing people’s resilience. Regarding the second hypothesis, the results showed that hope was directly and positively related to resilience. In line with the results of the present study, in the RIM, there was a positive and significant relationship between hope and resilience [19]. Moreover, in other studies hope predicted resilience [47, 48]. In explaining this finding, it can be said that hope as an internal force in the face of unpleasant events plays an essential role in empowering individuals in determining if it has positive and realistic goals and is a driving force that can manage the physical and psychosocial problems caused by the disease [49]. The findings of the present study and RIM indicated that having hope in difficult living conditions and hardships was an important factor in adapting and increasing tolerance in coping with the disease.

Regarding the third hypothesis, the results showed that uncertainty had a negative relationship with resilience. In RIM, with increasing uncertainty in adolescents with cancer, their level of resilience decreased [19]. In the research entitled “Uncertainty, mental health, and psycho-social adjustment in patients with multiple sclerosis”, the result indicated high levels of uncertainty in these patients and caused a decrease in psychological adjustment [50]. It seems that inadequate information and poor preparation for the management of the disease and its complications can prevent the patient from predicting changes and make the patient uncertain about the occurrence of psychological and physical problems in the future, and in turn reduces the level of resilience in individuals [26].

Defensive coping was another risk factor, and the findings showed that there was a negative relationship between defensive coping and resilience; however, the difference was not significant. In a study, the results showed that people who used defensive coping styles had lower resilience [19]. These results may indicate that thalassemia patients with severe negative symptoms probably feel unable to control stress and, therefore, tend to ignore their problems instead of using positive coping methods [23]. This deficiency makes it more difficult for patients with thalassemia to adapt to the situation, increase and maintain essential interpersonal relationships, or avoid a negative situation [51]. Defensive coping showed a positive and significant relationship with courageous coping and indirectly exhibited a positive and significant effect on resilience through courageous coping. According to the Resilience in Illness Model, it seems that whenever a person engages in emotional and avoidant coping, he/she tries to manage these emotional situations with courageous coping strategies; however, in the future, if this tense situation continues, the use of courageous coping strategies in people decreases [19]. This finding indicates that these coping strategies play vital roles in helping to achieve resilience. Defensive coping can be converted to courageous coping if individuals are able to expand other protective factors to buffer the risk factors [17]. In this study, social support and hope seemed to play a role in changing defensive coping to courageous coping.

Regarding mediating effects (fifth hypothesis), we found that courageous coping partially mediated the relationship between social support and resilience. In other words, social support showed a direct, positive, and significant relationship with resilience and was indirectly related to resilience through courageous coping. These findings are in line with the previous literature [14, 19, 52], In other studies, with the increase in social support, its effect on promoting adaptation methods increased and, in turn, the positive outcome of adaptation or resilience also increased; it is also one of the important predictors of resilience and has a positive effect on health and recovery [53, 54]. In other words, when people received enough social support, they were more likely to use positive coping strategies to achieve a higher level of resilience [55]. Shing et al. (2016) found that individuals who had higher resilience were able to recover from stressful situations faster, had a positive view of stressful situations, and were more involved in active coping behaviors [56]; those adolescents who received adequate social support from family, friends, and significant others could apply courageous coping which influenced them to carry out actions when faced with adversity [14]. These findings supported the hypotheses proposed by the RIM, i.e. it seems that received social support from family, friends, and significant others is very valuable in helping people deal with difficulties and unpleasant events adversities [18, 19]. Regarding courageous coping, as a mediator in the relationship between hope and resilience, the results showed that this relationship was fully mediated and confirmed the fifth hypothesis. People who had a high level of resilience in their lives had a positive approach to life and had higher hope and a high sense of self-sufficiency [19]. These findings were consistent with previous literature; people who have hopeful views and are, therefore, able to set goals, imagine alternative strategies to attain them, and demonstrate the ability to motivate themselves are likely to have higher levels of life satisfaction [57], resilience [58], and well-being [59]. This study has theoretical and practical implications. First, in the theoretical dimension, it recommended a mediation model, demonstrating that courageous coping mediated the relationship between protective and risk factors with resilience among adolescents with BTM. Second, in the practical level, health care professionals (i.e., nurses) should pay more attention to the role of protective factors in thalassemia patients, which can help reduce psychological distress and improve resilience. In addition, an effect of protective and risk factors on resilience passes through the path of courageous coping, so resilience interventions may be strengthened by clarifying the effects of these protective (hope, social support) and risk (defensive coping and uncertainty) factors on courageous coping. In other words, positive coping interventions should be planned to simultaneously enhance the level of protective factors; therefore, we can eventually promote resilience.

### Limitations and Strengths

There were several limitations in the present study. First, the participants in the present study were selected using the convenience sampling method. Therefore, the study sample may not be representative of all patients. However, we chose sampling from the largest thalassemia center in Southern Iran, and the results can be generalized to some extent. Second, mediation analysis with cross-sectional data cannot establish causality. Third, self-report data may be subject to social desirability bias. Finally, there are other crucial variables such as spirituality and characteristics related to the disease that our mediators may indirectly impact as well; however, due to considerations of time and the complexity of the model, we omitted these variables.

Despite these limitations, identifying risk and protective factors for resilience can provide valuable guidance for planning and designing nursing interventions for healthcare providers. In this study, the courageous coping strategy has played an important role in modulating the risk factors to increase resilience, and social support has an important role in promoting resilience. Therefore, designing interventions in this field can lead to the improvement of resilience. Another valuable consequence is the use of structural equation modeling, which has increased the power of the study in estimating the relationships between variables.

## Conclusion

Generally, the findings indicated the predictive roles of risk and protective factors among adolescents and young adults with BTM. Resilience is recognized as a critical concept in recovery from the long-term effects of an event in adolescents, but its protective and risk factors have not been sufficiently investigated. We found that social support, hope, uncertainty, and defensive coping affected resilience, and these factors had indirect effects on resilience through courageous coping. Based on these findings, health care professionals (i.e., nurses) should consider promoting intervention strategies to improve resilience through enhancing positive coping strategies in adolescent populations with BTM.

## Data Availability

The datasets generated and/or analysed during the current study are not publicly available due to the necessity to ensure participant confidentiality policies and laws of the country but are available from the corresponding author on reasonable request.

## Abbreviations

BTM: Beta-thalassemia major
RIM: Resilience in illness model
SEM: Structural equation modeling
CD-RISC: Connor–Davidson Resilience Scale
PSSS: Perceived Social Support Scale
JCS: The Jalowiec Coping Scale
HHI: Herth Hope Index
AMOS: Analysis of Moment Structures
